# Measurement of the total antioxidant response using a novel automated method in subjects with nonalcoholic steatohepatitis

**DOI:** 10.1186/1471-230X-5-35

**Published:** 2005-11-11

**Authors:** Mehmet Horoz, Cengiz Bolukbas, Fusun F Bolukbas, Tevfik Sabuncu, Mehmet Aslan, Serpil Sarifakiogullari, Necla Gunaydin, Ozcan Erel

**Affiliations:** 1 Harran University, Department of Internal Medicine, Sanliurfa, Turkey; 2 Harran University, Department of Gastroenterology, Sanliurfa, Turkey; 3 Harran University, Department of Endocrinology, Sanliurfa, Turkey; 4 Harran University, Department of Biochemistry, Sanliurfa, Turkey

## Abstract

**Background:**

Oxidative stress, an increase in oxidants and/or a decrease in antioxidant capacity, is one of the potential biochemical mechanisms involved in the pathogenesis of nonalcoholic steatohepatitis. We aimed to investigate the total antioxidant response using a novel automated method in nonalcoholic steatohepatitis subjects. As a reciprocal measure, we also aimed to determine total peroxide level in the same plasma samples.

**Methods:**

Twenty-two subjects with biopsy proven nonalcoholic steatohepatitis and 22 healthy controls were enrolled. Total antioxidant response and total peroxide level measurements were done in all participants. The ratio percentage of total peroxide level to total antioxidant response was regarded as oxidative stress index.

**Results:**

Total antioxidant response of subjects with nonalcoholic steatohepatitis was significantly lower than controls (p < 0.05), while mean total peroxide level and mean oxidative stress index were higher (all p < 0.05).

In subjects with nonalcoholic steatohepatitis, fibrosis score was significantly correlated with total peroxide level, total antioxidant response and oxidative stress index (p < 0.05, r = 0.607; p < 0.05, r = -0.506; p < 0.05, r = 0.728, respectively). However, no correlation was observed between necroimflamatory grade and those oxidative status parameters (all p > 0.05).

**Conclusion:**

Nonalcoholic steatohepatitis is associated with increased oxidant capacity, especially in the presence of liver fibrosis. The novel automated assay is a reliable and easily applicable method for total plasma antioxidant response measurement in nonalcoholic steatohepatitis.

## Background

Non-alcoholic fatty liver disease (NAFLD) is increasingly recognized as a significant cause of morbidity in developed countries. NAFLD represents a spectrum of liver disease ranging from bland steatosis to severe steatohepatitis. The prevalence of non-alcoholic steatohepatitis (NASH) is 2.1–6.3% in the general population, rising to 9–40% in obese individuals with a body mass index (BMI) of 30 kg/m^2 ^or more. Steatohepatitis can be progressive, cause fibrosis and cirrhosis, and can ultimately lead to liver failure and hepatocellular carcinoma in a minority of patients [1-4].

The pathogenesis of NASH remains unclear and the factors, which cause the progression from bland steatosis to steatohepatitis, often termed the 'second hit', remain poorly understood [5-7]. One potential biochemical mechanism of pathogenesis of NASH is oxidative stress [8,9]. Oxidative stress is a cardinal feature of alcoholic steatohepatitis [10]. Histological similarity between NASH and alcohol induced liver disease suggests some shared pathogenetic features such as oxidative stress and cytokine-mediated injury [5-8,11,12]. In addition, evidence of oxidative stress have been found in human livers showing steatosis or NASH [13], and in experimental models of NASH [14]. The efficacy of several antioxidant agents on hepatic steatosis, inflammation, and fibrosis in subjects with NASH has been investigated in several small, open-label studies [15-17]. In all of these studies, those antioxidant agents exerted beneficial effects in improving necroinflammatory activity or fibrosis or both. These findings are also suggestive for the role of oxidative stress in the pathogenesis of NASH.

Various methods have been developed for the measurement of total antioxidant status. However, there is not yet an accepted "gold standard" reference method [18], and decisions concerning standardization, and the terms and units used for the measurement of total antioxidant response (TAR) [19]. This implies that this topic needs to be studied further [20].

In the present study, we aimed to measure TAR using a novel automated method in NASH subjects [20]. As a reciprocal measure, the total peroxide levels were also measured at the same plasma samples. The ratio percentage of the total plasma peroxide level to the plasma TAR value was regarded as oxidative stress index (OSI) [21].

## Methods

### Enrollment of patients

Twenty-two subjects with biopsy proven NASH (19 male, 3 female; mean age, 37.7 ± 8.8) and 22 healthy controls (17 male, 5 female; mean age, 34.6 ± 9.3) were enrolled in the present study. The study protocol was approved by the local research committee for ethics. All subjects were informed about the study and the written consent was obtained from each one.

### Initial evaluation

The major indications for liver biopsy in those 22 subjects were ultrasonographically diagnosed fatty liver and elevation in alanine aminotransferase (ALT).

Diagnosis of NASH was made according to the following criteria:

- Existence of hepatic steatosis (≥10% of hepatocytes affected) with acinar zone 3 hepatocellular injury (ballooning degeneration) and/or lobular inflammation, with or without Mallory's hyaline and pericellular and/or sinusoidal fibrosis, on liver byopsy, which was performed within the previous 12 months. Necroimflamatory grading and fibrosis scoring were based on a modification of the scoring system proposed by Brunt et al [3].

- Negative serological markers for viral infection such as HBsAg, anti-HCV or anti-HIV, and immunological disorders such as antinuclear anti-bodies, anti-smooth muscle antibodies, and anti-liver/kidney microsomes type 1 antibody.

- No evidence that favor of metabolic liver disease such as Wilson's disease and hemochromatosis and alpha-1 antitrypsin deficiency.

Necroimflamatory grades and fibrosis scores of the 22 subjects with NASH were as follows:

- Grade 1 (mild) necroimflamatory changes, in 5 (22.73%) subjects; grade 2 necroimflamatory changes (moderate), in 12 (54.54%) subjects; grade 3 necroimflamatory changes, in 5 (22.73%) subjects.

- Stage 0 Zone 3 perisinusoidal/pericellular fibrosis, in 2 (9.1%) subjects; stage 1, in 9 (40.9%) subjects; stage 2, in 9 (40.9%) subjects; stage 3, in 2 (9.1%) subjects.

- None of the subjects with NASH had stage 4 fibrosis.

### Exclusion criteria

Exclusion criteria included recent gastrointestinal by-pass surgery, pregnancy, serum total bilirubin level higher than 2 mg/dL, usage of esterogens, tamoxifen, glucocoticoids, usage of supplemental vitamins, calcium-channel blockers, aspirin, amiodarone, and methotrexate in the previous 6 months, existence of diabetes mellitus, coronary artery disease, rheumatoid arthritis, cancer, systemic or local infection, and history of excess alcohol ingestion, averaging more than 30 gm/day (3 drinks per day) in the previous 10 years, or history of alcohol intake averaging greater than 10 gm/day (1 drink per day: 7 drinks per week) in the previous 1 year.

Recently, it has been suggested that oxidative stress itself may play a crucial role in pathogenesis of diabetes mellitus [22]. Additionally, both increase in oxidative stress and decrease in antioxidant capacities could be related to the complications of diabetes mellitus [23]. Thus, in the present study, we excluded the subjects with diabetes mellitus in order to reflect the accurate role of oxidative stress in pathogenesis NASH.

The clinical and demographic data of the study groups were shown in Table 1.

### Blood collection

Blood samples were obtained in fasting state. Samples were withdrawn from a cubital vein into heparinised tubes and immediately stored on ice at 4°C. Then, the plasma was separated from the cells by centrifugation at 3000 rpm for 10 min, and the plasma samples were stored at -80°C until analysis.

### Measurement of the total antioxidant status of plasma

The total antioxidant status of the plasma was measured using a novel automated colorimetric measurement method for TAR developed by Erel [20]. In this method, the hydroxyl radical, the most potent biological radical, is produced by the Fenton reaction, and reacts with the colourless substrate O-dianisidine to produce the dianisyl radical, which is bright yellowish-brown in colour. Upon the addition of a plasma sample, the oxidative reactions initiated by the hydroxyl radicals present in the reaction mix are suppressed by the antioxidant components of the plasma, preventing the colour change and thereby providing an effective measure of the total antioxidant capacity of the plasma. The assay results are expressed as mmol Trolox eq./L, and the precision of this assay is excellent, being lower than 3% [24].

### Measurement of total plasma peroxide concentration

The total plasma peroxide concentrations were determined using the FOX_2 _method [25] with minor modifications [21]. The FOX_2 _test system is based on the oxidation of ferrous iron to ferric iron by the various types of peroxides contained in the plasma samples, in the presence of xylenol orange which produces a coloured ferric-xylenol orange complex whose absorbance can be measured. The FOX2 reagent was prepared by dissolving ammonium ferrous sulphate (9.8 mg) in 250 mM H_2_SO_4 _(10 ml) to give a final concentration of 250 mM ferrous iron in acid. This solution was then added to 90 ml HPLC-grade methanol containing 79.2 mg butylated hydroxytoluene (BHT). Finally, 7.6 mg xylenol orange was added, with stirring, to make the working reagent (250 mM ammonium ferrous sulphate, 100 mM xylenol orange, 25 mM H_2_SO_4_, and 4 nM BHT, in 90% (v/v) methanol in a final volume of 100 ml). The blank reagent contained all the components of the solution except ferrous sulphate. Aliquots (200 mL) of plasma were mixed with 1.8 ml FOX_2 _reagent. After incubation at room temparature for 30 min, the vials were centrifuged at 12,000 g for 10 min. The absorbance of the supernatant was then determined at 560 nm. The total peroxide content of the plasma samples was determined as a function of the difference in absorbance between the test and blank samples using a solution of H_2_O_2 _as standard. The coefficient of variation for individual plasma samples was less than 5%.

### Oxidative stress index

The ratio percentage of the total peroxide to the total anti-oxidant potential gave the oxidative stress index, an indicator of the degree of oxidative stress [21].

### Statistical analysis

Data were presented as mean ± SD. Continuous variables were compared using Student t test. Qualitative variables were tested by Chi-square test. Spearman correlation test was used to find out the correlation of fibrosis scores and necroinflamatory grades with total preroxide level, OSI or TAR. Correlations of serum triglyceride and cholesterol levels with total peroxide level, OSI or TAR were analyzed using Pearson correlation test. p < 0,05 was considered as statistical significance.

## Results

Two groups were comparable for age, BMI, gender distribution and smoking habit (p value was >0.05 for each comparison). Serum triglyceride and cholesterol levels were significantly higher in subjects with NASH than controls (p value was >0.05 for each comparison).

TAR was 0.85 + 0.11 and 1.88 + 0.32 mmol Trolox eq./L in subjects with NASH and controls, respectively (p < 0.05). Total plasma peroxide level of subjects with NASH and controls was 53.3 + 7.7 and 33.0 + 14.2 μmol H_2_O_2_/L, respectively (p < 0.05). OSI was 0.64 + 0.14 and 0.19 + 0.11 AU in subjects with NASH and controls, respectively (p < 0.05).

Total peroxide level and OSI were positively correlated with serum triglyceride level in subjects with NASH (p < 0.05, r = 0.432; p < 0.05, r = 0.521, respectively), while no correlation with serum cholesterol level (p > 0.05 for each comparison). Serum triglyceride and cholesterol levels were not correlated with both total peroxide level and OSI value in control subjects (p > 0.05 for each comparison). No correlation was observed between TAR, and serum triglyceride and cholesterol level in both NASH subjects and controls (p > 0.05 for each comparison).

**Figure 1:**
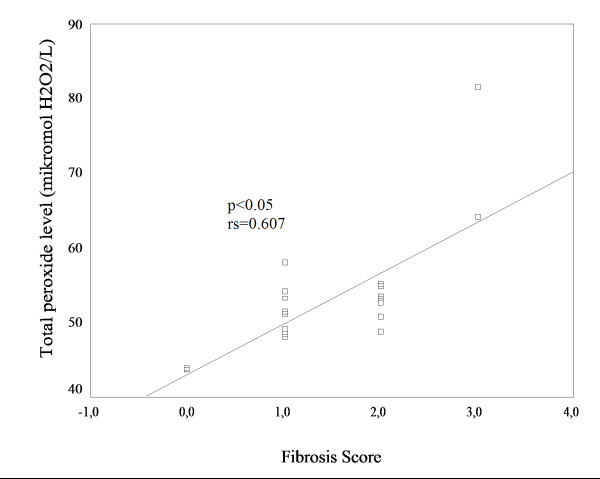
Correlation between serum total peroxide levels and the score of liver fibrosis among nonalcoholic steatohepatitis group.

**Figure 2:**
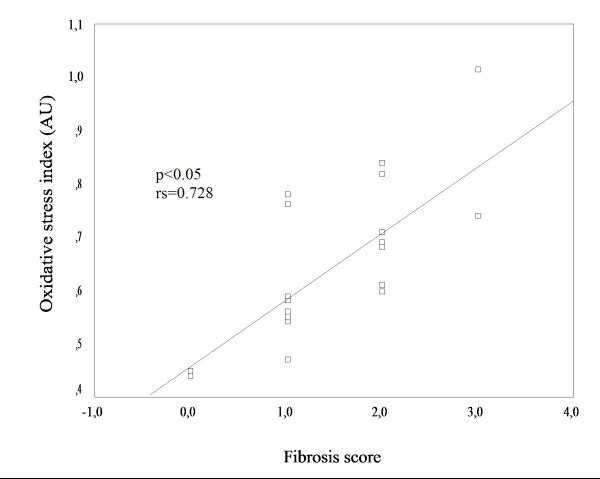
Correlation between oxidative stress index and the score of liver fibrosis among nonalcoholic steatohepatitis group. AU, arbitrary unit.

**Figure 3:**
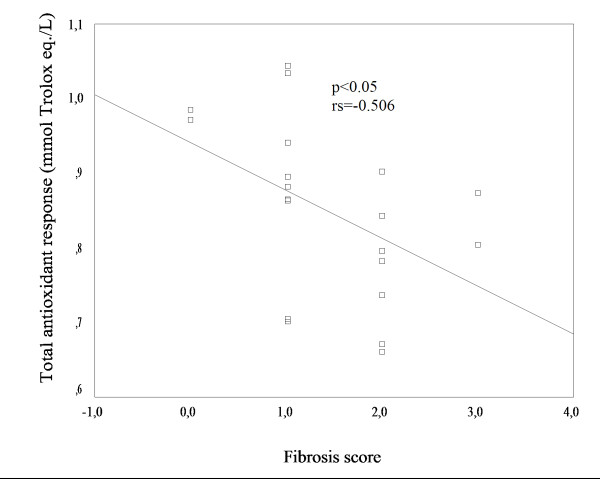
Correlation between serum total antioxidant response and the score of liver fibrosis among nonalcoholic steatohepatitis group.

Fibrosis scores of subjects with NASH were positively correlated with total peroxide level and OSI (p < 0.05, r = 0.607 and p < 0.05, r = 0.728, respectively) (Fig 1, 2), while negatively correlated with TAR (p < 0.05, r = -0.506) (Fig 3). No significant correlation was observed between necroimflamatory grades, and total peroxide level, TAR and OSI (p value was >0.05 for each comparison).

## Discussion

Steatosis of the liver is mediated by a multiple factors such as increase in dietary fat intake, release of free fatty acids (FFA) from adipose tissue, insufficient hepatic lipid secretion, and development of insulin resistance [26]. FFA oxidation, CYP2E1 induction, leukocyte infiltration and activation of NADPH oxidase, and mitochodrial dysfunction involving electron transfer inhibition in the respiratory chain increase the production of reactive oxygen species (ROS) such as singlet oxygen, superoxide anion, hydrogen peroxide, and hydroxyl radical [10].

In the present study, in subjects with NASH, significant increases in both total peroxide level and OSI were accompanied with significant decreases in TAR compared to control subjects. In the presence of increased total peroxide level, a decrease in TAR indicates to a high degree of oxidative stress. In addition, in subjects with NASH, total peroxide level and OSI showed positive correlation with fibrosis score. In contrast, TAR showed negative correlation with TAR. On the basis of these findings, it can also be suggested that oxidative stress may have a role in the progression of steatohepatitis to liver fibrosis.

In order to reflect the true state of oxidative stress in the liver, it is more ideal to measure lipid peroxidation markers and antioxidant components in hepatic tissue. Nevertheless, it is impossible to perform multiple tests on very limited amounts of biopsy specimen that obtained in needle biopsy. In addition, liver biopsy carries a significant morbidity and even mortality risk. It is not an ethical approach to perform liver biopsy in healthy controls to provide a comparison with NASH subjects. Thus, in the present study, we have chosen to measure TAR and total peroxide level in plasma samples.

In a few studies, in NASH subjects, antioxidant and oxidant capacities have been investigated [27-29]. Koruk et al [27] reported an increase in oxidative stress in NASH subjects. However, in their study, plasma anitoxidant capacities observed to be comparable in NASH subjects and controls. They suggested that impaired antioxidant defense mechanisms in responding to increased oxidative stress might be an important factor in the pathogenesis of NASH. In a study of Fierbinteanu-Braticevici et al [28], serum index of oxidative stress have been suggested as an independent risk factor for fibrosis in the course of NASH.

In a recent study, which was conducted by Videla et al [29], it has been shown that NAFLD patients with steatosis exhibit a substantial pro-oxidant condition in the liver at early stages of steatosis. This pro-oxidant condition was observed to occur concomitantly with a significant decrease in hepatic SOD activity, changes involving an overall derangement in the antioxidant status of the liver, with the consequent diminution in the antioxidant capacity of plasma. They also observed that further exacerbation in oxidative stress was associated with CYP2E1 induction in patients with steatohepatitis.

Various antioxidants have additive effects on oxidative status. In human serum, the cooperation of these antioxidants protects the organism against the attacks by free radicals [30]. Although the concentration of plasma antioxidant components can be measured individually, these measurements may be time- and cost-consuming and labour intensive. In addition, it may not accurately reflect the total antioxidant status [24]. Thus, the accurate antioxidant capacity of the organism can only be determined by the measurement of TAR [19,20].

In concordance with the study of Videla et al [29], in the present study, we determined the antioxidant capacity using TAR measurement. The oxidants and antioxidant capacity were determined simultaneously to evaluate oxidative stress accurately. However, in study of Videla et al. [29], TAR measurement was performed using ferric reducing ability of plasma (FRAP), while a novel automated method in our study.

The most widely used methods for TAR measurement are colorimetric, fluorescence, and chemiluminescence [31-33]. The fluorescence and chemiluminescence methods need sophisticated techniques and, in most routine clinical biochemistry laboratories, these improved systems are not present. Randox- total antioxidant status (TAS) assay and FRAP assay are the widely used colorimetric TAR measurement methods. In the FRAP assay, the reference range of serum TAR is lowest because this assay practically measures non-protein total antioxidant capacity. However, proteins constitute the main antioxidant component of serum (plasma). The Randox-TAS assay can determine the antioxidative effects of bilirubin, vitamin C, uric acid, polyphenols, and proteins; hence, the reference range for serum TAR is higher than that for the FRAP assay. The novel method is more sensitive for determining the antioxidative effects of bilirubin, uric acid, vitamin C, polyphenols, and proteins. Hence, serum TAR measured by the novel method is higher than those of the Randox-TAS and FRAP assays [20].

In addition, the novel method, which was used in the present study, provides further several advantages in comparison with other currently available methods. It is simple and cheap, and can easily be fully automated. It also does not interact with commonly occurring serum components such as bilirubin, serum lipids, and anticoagulants. Accurate measurements of TAR can be obtained within approximately 10 minutes, making this assay eminently suitable for the clinical biochemistry laboratory [20].

## Conclusion

NASH is associated with increased oxidant capacity, especially in the presence of liver fibrosis. The novel automated calorimetric assay is a useful, reliable, simple and easily applicable method in the assessment of the total plasma antioxidant response in NASH.

## Competing interests

The author(s) declare that they have no competing intersts.

## Authors' contributions

MH conceived the study, and participated in its design and coordination, and in the sequence alignment and drafted the manuscript. CB conceived the study, and participated in its design and coordination and in the sequence alignment, and drafted the manuscript.

FFB conceived the study, and participated in the sequence alignment and drafted the manuscript. TS conceived the study, and participated in its design and coordination. MA conceived the study, and participated in its design and coordination, and collected the samples. SS conceived the study, and participated in its design and coordination, and collected the samples NG conceived the study, collected the samples and carried out the laboratory analysis. OE conceived the study, and participated in its design and coordination, and carried out the laboratory analysis.

## Pre-publication history

The pre-publication history for this paper can be accessed here:



## Figures and Tables

**Table 1:** The clinical and demographic data of the study groups

	Subjects with NASH	Control subjects
Age	37.7 ± 8.8	34.6 ± 9.3
Weight	83.5 ± 9.4	80.7 ± 14.5
Height	171.1 ± 5.8	169 ± 7.8
BMI	28.9 ± 3	27.7 ± 3.3
Triglyceride	228 ± 93.7*	159.8 ± 62.6
Cholesterol	205.4 ± 44.4*	168.5 ± 35.1
ALT	70 ± 29	23 ± 6
Smoking habit	14 (63.6%)	11 (50%)

Data were presented as mean ± SD.

*p<0.05 vs. controls.

ALT, Alanine Aminotransferase; BMI, Body Mass Index.

## References

[B1] Angulo P (2002). Nonalcoholic fatty liver disease. N Engl J Med.

[B2] Neuschwander-Tetri BA, Caldwell SH (2003). Nonalcoholic steatohepatitis: summary of an AASLD Single Topic Conference. Hepatology.

[B3] Brunt EM, Janney CG, Di Bisceglie AM, Neuschwander-Tetri BA, Bacon BR (1999). Nonalcoholic steatohepatitis: a proposal for grading and staging the histological lesions. Am J Gastroenterol.

[B4] Matteoni CA, Younossi ZM, Gramlich T, Boparai N, Liu YC, McCullough AJ (1999). Nonalcoholic fatty liver disease: a spectrum of clinical and pathological severity. Gastroenterology.

[B5] Caldwell SH, Oelsner DH, Iezzoni JC, Hespenheide EE, Battle EH, Driscoll CJ (1999). Cryptogenic cirrhosis: clinical characterization and risk factors for underlying disease. Hepatology.

[B6] Green RM (2003). NASH – hepatic metabolism and not simply the metabolic syndrome. Hepatology.

[B7] Yang SQ, Lin HZ, Lane MD, Clemens M, Diehl AM (1997). Obesity increases sensitivity to endotoxin liver injury: implications for the pathogenesis of steatohepatitis. Proc Natl Acad Sci U S A.

[B8] Chitturi S, Farrell GC (2001). Etiopathogenesis of nonalcoholic steatohepatitis. Semin Liver Dis.

[B9] Day CP, James OF (1998). Steatohepatitis: a tale of two "hits"?. Gastroenterology.

[B10] Videla LA, Rodrigo RN, Araya J, Poniachik J (2004). Oxidative stress and depletion of hepatic long-chain polyunsaturated fatty acids may contribute to nonalcoholic fatty liver disease. Free Radic Biol Med.

[B11] Su GL (2002). Lipopolysaccharides in liver injury: molecular mechanisms of Kupffer cell activation. Am J Physiol Gastrointest Liver Physiol.

[B12] Hoek JB, Pastorino JG (2002). Ethanol, oxidative stress, and cytokine-induced liver cell injury. Alcohol.

[B13] Bhat VB, Madyastha KM (2001). Antioxidant and radical scavenging properties of 8-oxo derivatives of xanthine drugs pentoxifylline and lisofylline. Biochem Biophys Res Commun.

[B14] Leclercq IA, Farrell GC, Field J, Bell DR, Gonzalez FJ, Robertson GR (2000). CYP2E1 and CYP4A as microsomal catalysts of lipid peroxides in murine nonalcoholic steatohepatitis. J Clin Invest.

[B15] Hasegawa T, Yoneda M, Nakamura K, Makino I, Terano A (2001). Plasma transforming growth factor-beta1 level and efficacy of alpha-tocopherol in patients with non-alcoholic steatohepatitis: a pilot study. Aliment Pharmacol Ther.

[B16] Sanyal AJ, Mofrad PS, Contos MJ, Sargeant C, Luketic VA, Sterling RK, Stravitz RT, Shiffman ML, Clore J, Mills AS (2004). A pilot study of vitamin E versus vitamin E and pioglitazone for the treatment of nonalcoholic steatohepatitis. Clin Gastroenterol Hepatol.

[B17] Harrison SA, Torgerson S, Hayashi P, Ward J, Schenker S (2003). Vitamin E and vitamin C treatment improves fibrosis in patients with nonalcoholic steatohepatitis. Am J Gastroenterol.

[B18] Hubel CA (1999). Oxidative stress in the pathogenesis of preeclampsia. Proc Soc Exp Biol Med.

[B19] Erel O (2004). A novel automated direct measurement method for total antioxidant capacity using a new generation, more stable ABTS radical cation. Clin Biochem.

[B20] Erel O (2004). A novel automated method to measure total antioxidant response against potent free radical reactions. Clin Biochem.

[B21] Harma M, Harma M, Erel O (2003). Increased oxidative stress in patients with hydatidiform mole. Swiss Med Wkly.

[B22] Davi G, Falco A, Patrono C (2005). Lipid peroxidation in diabetes mellitus. Antioxid Redox Signal.

[B23] Gil-Del Valle L, Milian Lde L, Toledo A, Vilaro N, Tapanes R, Otero MA (2005). Altered redox status in patients with Diabetes Mellitus type I. Pharmacol Res.

[B24] Cao G, Prior RL (1998). Comparison of different analytical methods for assessing total antioxidant capacity of human serum. Clin Chem.

[B25] Miyazawa T (1989). Determination of phospholipid hydroperoxides in human blood plasma by a chemiluminescence-HPLC assay. Free Radic Biol Med.

[B26] Te Sligte K, Bourass I, Sels JP, Driessen A, Stockbrugger RW, Koek GH (2004). Non-alcoholic steatohepatitis: review of a growing medical problem. Eur J Intern Med.

[B27] Koruk M, Taysi S, Savas MC, Yilmaz O, Akcay F, Karakok M (2004). Oxidative stress and enzymatic antioxidant status in patients with nonalcoholic steatohepatitis. Ann Clin Lab Sci.

[B28] Fierbinteanu-Braticevici C, Bengus A, Neamtu M, Usvat R (2002). The risk factors of fibrosis in nonalcoholic steatohepatitis. Rom J Intern Med.

[B29] Videla LA, Rodrigo R, Orellana M, Fernandez V, Tapia G, Quinones L, Varela N, Contreras J, Lazarte R, Csendes A, Rojas J, Maluenda F, Burdilas P, Diaz JC, Smok G, Thielemann L, Poniachik J (2004). Oxidative stress-related parameters in the liver of non-alcoholic fatty liver disease patients. Clin Sci (Lond).

[B30] Wayner DD, Burton GW, Ingold KU, Barclay LR, Locke SJ (1987). The relative contributions of vitamin E, urate, ascorbate and proteins to the total peroxyl radical-trapping antioxidant activity of human blood plasma. Biochim Biophys Acta.

[B31] Janaszewska A, Bartosz G (2002). Assay of total antioxidant capacity: comparison of four methods as applied to human blood plasma. Scand J Clin Lab Invest.

[B32] Prior RL, Cao G (1999). In vivo total antioxidant capacity: comparison of different analytical methods. Free Radic Biol Med.

[B33] Schlesier K, Harwat M, Bohm V, Bitsch R (2002). Assessment of antioxidant activity by using different in vitro methods. Free Radic Res.

